# The Role of Molecular Autopsy in Concealed Cardiomyopathies

**DOI:** 10.3390/genes16111273

**Published:** 2025-10-28

**Authors:** Oscar Campuzano, Coloma Tirón, Estefanía Martínez-Barrios, Andrea Greco, Jose Cruzalegui, Fredy Chipa, Sergi Cesar, Erika Fernanda Merchan, Mónica Coll, Anna Fernández-Falgueras, Ramon Brugada, Marisa Ortega, Núria Molina, Eneko Barberia, Rocío Toro, Antonio Oliva, Simone Grassi, Georgia Sarquella-Brugada

**Affiliations:** 1 Medical Science Department, School of Medicine, University of Girona, 17003 Girona, Spain; georgia@brugada.org; 2 Institut Investigacions Biomèdiques de Girona, IDIBGI, 17190 Girona, Spain; ctiron.girona.ics@gencat.cat (C.T.); monicacoll.girona.ics@gencat.cat (M.C.); annafernandez.girona.ics@gencat.cat (A.F.-F.); rbrugada@idibgi.org (R.B.); 3 Centro de Investigación Biomédica en Red, Enfermedades Cardiovasculares, 28029 Madrid, Spain; 4 Unitat de Genòmica i Medicina Personalitzada, Laboratori Clínic Territorial, Institut Català de la Salut, 17003 Girona, Spain; 5 Inherited Cardiac Diseases Unit, Department of Cardiology, Hospital Universitari Dr Josep Trueta, 17007 Girona, Spain; 6 Pediatric Arrhythmias, Inherited Cardiac Diseases and Sudden Death Unit, Cardiology Department, Hospital Sant Joan de Déu, Esplugues de Llobregat, 08950 Barcelona, Spain; estefania.martinez@sjd.es (E.M.-B.); andrea.greco@sjd.es (A.G.); josecarlos.cruzalegui@sjd.es (J.C.); fredy.chipa@sjd.es (F.C.); sergio.cesar@sjd.es (S.C.); erikafernandamp@gmail.com (E.F.M.); 7 Arrítmies Pediàtriques, Cardiologia Genètica i Mort Sobtada, Malalties Cardiovasculars en el Desenvolupament, Institut de Recerca Sant Joan de Déu, Esplugues de Llobregat, 08950 Barcelona, Spain; 8 European Reference Network for Rare, Low Prevalence and Complex Diseases of the Heart (ERN GUARD-Heart), 1105 AZ Amsterdam, The Netherlands; 9 Pediatrics Department, School of Medicine, University of Barcelona, 08036 Barcelona, Spain; 10 Forensic Pathology Service, Institut de Medicina Legal i Ciències Forenses de Catalunya, 08075 Barcelona, Spain; mlortegasan@xij.gencat.cat (M.O.); nuriamolina@xij.gencat.cat (N.M.); eneko.barberia@xij.gencat.cat (E.B.); 11 Faculty of Medicine, University of Vic—Central University of Catalonia (UVic-UCC), 08500 Vic, Spain; 12 Faculty of Medicine and Health Sciences, University of Rovira and Virgili, 43201 Reus, Spain; 13 Medicine Department, School of Medicine, University of Cádiz, 11003 Cádiz, Spain; rocio.toro@uca.es; 14 Area of Pathology, Department of Woman and Child Health and Public Health, Fondazione Policlinico Universitario A. Gemelli IRCCS, 00168 Rome, Italy; antonio.oliva@unicatt.it; 15 Department of Health Sciences, Section of Forensic Medical Sciences, University of Florence, 50134 Florence, Italy; simone.grassi@unifi.it

**Keywords:** molecular autopsy, genetics, concealed cardiomyopathies, arrhythmias, sudden cardiac death, forensics

## Abstract

A conclusive and early diagnosis of cardiomyopathy is essential for implementing preventive therapeutic measures and, therefore, reducing the risk of malignant arrhythmias and even sudden cardiac death. Occasionally, this lethal event can be the first manifestation of cardiomyopathy, with or without a clear structural defect. In cases of sudden death, especially in young patients, the autopsy may be ambiguous and therefore lack a definitive diagnosis of cardiomyopathy, although it can sometimes identify signs that lead us to suspect it. This is one of the current challenges of forensic science, where occult cardiomyopathies often remain unidentified without additional testing that is not routinely included in current forensic protocols. In this protocol, it is crucial to perform a molecular autopsy but also to include additional data, especially family history, that will help conclude or at least suspect this entity. Obtaining this diagnosis or suspicion of concealed cardiomyopathy not only provides an answer to the unexpected death but also helps the relatives determine the cause of death. In addition, physicians should initiate a family assessment to identify other family members who may be at risk early and adopt personalized preventive measures.

## 1. Introduction

The condition known as “concealed cardiomyopathy” (CC) refers to a sudden death (SD) with a forensic autopsy that does not identify conclusive cardiac abnormalities (neither microscopic nor macroscopic) but postmortem genetic studies (known as molecular autopsy, MA) identify a deleterious alteration in a gene associated with cardiomyopathy. These findings highlight a phase of malignant arrhythmias prior to the structural phenotype of cardiomyopathy or ultrastructural changes in the myocardium, with this electrical alteration being responsible for a recovered sudden cardiac arrest/or sudden cardiac death (SCA/SCD) [1]. In forensic medicine, the cause of SD remains unexplained (SUD) in 5–10% of cases due to the absence of cardiac structural abnormalities after a complete forensic autopsy [2,3]. In the young population, called sudden unexplained death in the young (SUDY), the number increases up to 30–40% [4]. In these young cases, almost 50% of SUD show unknown previous pathology and a normal heart, suggesting a malignant arrhythmia as the most likely cause of decease [5].

The first studies suggesting the phenomenon of CC leading to SCA/SCD in an apparently normal heart were reported more than 15 years ago [6,7]. Both studies focused on postmortem cases showing minor tissue changes characteristic of the early stages of what was then called arrhythmogenic right ventricular cardiomyopathy (ARVC) despite not showing a definite diagnosis. All of these cases carried deleterious genetic variants in one of the genes associated with this cardiac condition, suggesting that ARVC is a progressive entity in which electrical disturbances can often manifest without the replacement of myocardial fibrofatty tissue. This has been confirmed by more recent studies focusing on this cardiac entity [8], as well as in other cardiomyopathies [9,10]. To date, there are few studies analyzing CC in cases of SCA/SUD with an inconclusive autopsy. The most common uncertain findings in those studies were left ventricular hypertrophy without disarray, idiopathic left ventricular dilatation, and unspecific fibrosis of the myocardium. These findings may be innocent bystanders or truly involved in SCD as a partial phenotypic expression of an underlying cardiomyopathy. Despite differences in autopsy protocols, age of cohorts, and genetic analysis, the rate ranges from 2% to 18% [5,10,11,12,13,14]. Taking all these aspects into account, we conducted this review to delve deeper into the role of MA when SUDY cases occur and CC is suspected.

To search the data, a systematic literature revision was conducted in PubMed, Web of Science, Scopus, and Embase databases for studies published until August 2025. We included items concerning “Autopsy”, Molecular autopsy”, “Genetics”, “Concealed cardiomyopathies”, “Cardiomyopathies”, “Inherited arrhythmogenic syndromes”, “Arrhythmias”, “Cardiac channelopathies”, “Sudden cardiac death”, “Cardiac pathologist” and “Forensics”. In our analysis we included only original English research articles and systematic reviews focused on the field.

## 2. Forensic Autopsy

**Figure 1:**
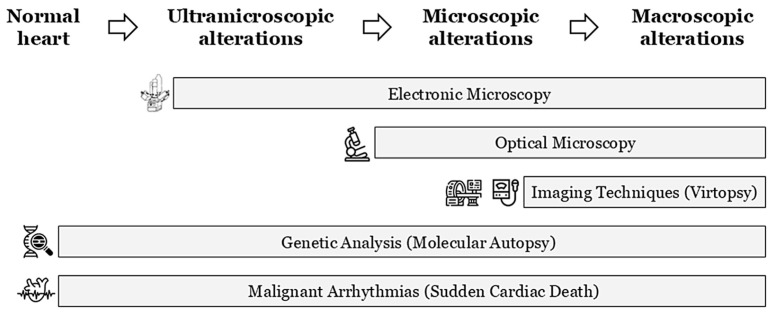
Progressive temporal evolution of cardiomyopathies. Identification techniques depending on the degree of tissue damage. Lethal arrhythmias may appear at any time.

During a forensic autopsy in cases of SCA/SCD, current international guidelines recommend a comprehensive cardiac examination; this should include both a macroscopic and microscopic evaluation of cardiac tissues to identify structural abnormalities or underlying pathologies that may have contributed to the fatal event [15,16]. Indeed, in the analysis of particularly complex cases and under optimal circumstances, the autopsy itself should be performed by or with the assistance of an expert in cardiac pathology and even the opinion of an expert centre should be sought if there is any doubt about the diagnosis [17]. In recent years, advanced imaging techniques, including virtual autopsy (virtopsy), have been incorporated into postmortem investigations. It helps to unravel any structural alterations that lead to the diagnosis of cardiomyopathy, although these methods are not yet mandatory in current protocols (Figure 1) [18]. Despite that the most common virtopsy technique is computed tomography, in cases of SUDY, magnetic resonance imaging should be preferred, because of the better tissue details. However, in clinical settings, the sensitivity of magnetic resonance for cardiomyopathies is relatively low and is even lower in the postmortem because of the impossibility of using a contrast medium that would help to detect features like fibrosis that can guide heart sampling. Therefore, for suspected cardiomyopathies, due to their progressive evolution over time, electron microscopy has been proposed to detect early signs of structural alteration. This technique can reveal ultramicroscopic changes that conventional imaging techniques cannot detect due to resolution limitations (Figure 1). Despite this limitation, ultramicroscopy procedures have not been included in routine autopsy protocols due to the technical difficulty of the process and the time required to process the results [19]. Finally, the results of various studies conducted in recent years have allowed postmortem genetic analysis to be incorporated into standard autopsies when routine protocols fail to determine a cause of the SUD, especially in young populations [20]. As mentioned above, cardiomyopathies are progressive diseases, and in the early stages, only ultramicroscopic or microscopic alterations can be identified using appropriate techniques (electronic or optical microscopy, respectively). As the disease progresses, microscopic effects can be observed at the macroscopic level, and imaging techniques can identify them to aid diagnosis (including virtopsy in postmortem cases). This temporal progression may be faster or slower depending on the type of alteration, gene, and external factors. The pathophysiological pathways implicated in the specific time related to progressive evolution of the disease has not yet been fully clarified (Table 1). At any of these stages, malignant arrhythmia can occur, leading to SUDY episodes if they occur in the earliest stages of the disease (Figure 1). To our knowledge, no studies have been published including the different protocols for intervention in Europe versus North America. In fact, forensic protocols for intervention may vary from country to country due to legal restrictions or economic costs, among other factors.

## 3. Molecular Autopsy

The MA process was first described almost 25 years ago and refers to a genetic test performed on postmortem samples from patients who died suddenly and who remained without a defined cause of death after a complete forensic autopsy [21]. Consequently, MA is currently widely recommended in cases of SUD without a definitive cause of death identified after conventional autopsy, aiming to identify the genetic alteration responsible for the unexpected and unexplained death [22,23]. In these cases, a cardiac channelopathy (also called inherited arrhythmogenic syndromes—IASs) or CC may be the first plausible cause of the suspected malignant arrhythmia that caused the SUD. In consequence, genes associated with IASs, as well as familial cardiomyopathies, should be analyzed [2,3,20]. The applicability of MA has progressively increased in recent years, assisting forensic pathologists in determine the cause of SUD and helping clinicians improve personalized care for family members [22,23]. Samples for MA can be obtained from blood, fresh tissue, or even formalin-fixed, paraffin-embedded (FFPE) samples. In cases of blood, it is recommended to use at least 5–10 mL stored in Ethylene Diamine Tetra Acetic acid (EDTA) tubes, within 48 h postmortem and kept refrigerated. In the case of fresh tissue, at least 5 g (mainly heart, liver, skeletal muscle, or spleen) should be kept frozen [15,16]. Although successful results are widely accepted, its application in forensic protocols is not yet routinely performed in developing countries, primarily due to economic cost. However, in developed countries, the challenge is often obtaining administrative/judicial permission, depending on the laws of each country [24]. Recent studies have reported postmortem genetic testing in no more than 40% of SUD cases with a suspected arrhythmic cause [22,25].

The progressive use of MA and increased genetic yield has been due to improvements in genetic sequencing technology and data processing. Today, a whole exome sequencing (WES) or a whole genome sequencing (WGS) are rapid, reliable, and cost-effective procedures in autopsy [26]. Therefore, panel selection is a critical factor in MA, because expanding the number of genes increases the chances of identifying clinically relevant variants but also raises the proportion of VUS without clear clinical consequences [27]. MA first focuses on the conventional cardiac ion channel, identifying up to 30% of rare variants with a potential deleterious role in these purely arrhythmogenic genes. However, a significant percentage of cases remained without any genetic alteration explaining the unexpected decease [20]. The expansion of the genes analyzed in MA to include genes for IAS and cardiomyopathies has increased diagnostic rates; this has been due to the identification of rare variants with potentially deleterious roles in some of the genes encoding structural proteins, thus unmasking a possible underlying cardiomyopathy in its early stages [13,28,29]. In recent years, MA in cases of normal or autopsies with unspecific findings have shown nearly 10% of definite pathogenic or likely pathogenic (P/LP) variants, up to 75% in genes associated with cardiomyopathy (*PKP2, FLNC, LMNA, MYBPC3,* and *TTN*), and the remaining 25% in genes associated with IAS (*KCNH2, KCNQ1*, and *RYR2*) [30,31]. This diagnostic yield in genes associated with cardiomyopathies is higher in postmortem cases with subdiagnostic findings at autopsy compared with cases showing a completely normal heart [10]. In fact, the diagnostic yield in cases of SUD with some structural findings identified during autopsy and suggestive of cardiomyopathy is similar to the genetic yield in patients diagnosed with any cardiomyopathy [12,32].

It is important to recognize the phenotypic overlap among genes implicated in various cardiomyopathies requiring the accurate interpretation of the specific variant rather than solely being gene involved. This is particularly critical in cases of SUD, where limited clinical and pathological data often hinder precise genotype–phenotype correlation [26]. Furthermore, some of these genes are often associated with malignant arrhythmias in the early stages of cardiomyopathy, with minimal or absent structural alterations (or even none or undetectable by routine tests at the clinical and forensic levels), as in cases of CC (Table 1). Therefore, despite massive sequencing, virtual gene panels including only genes with a definite clinical association are used in diagnosis. This technical approach ensures that the focus remains on clinically actionable genes and minimizes the burden of variants of uncertain significance (VUS) with no conclusive role in clinical translation so far [33,34]. Interpretation frameworks also pose a challenge, as many variants remain classified as VUS due to insufficient functional or population frequency data, underscoring the need for periodic updates and multidisciplinary consensus in genetic and clinical management. Since their role may change over time as new data emerge, recent studies recommend the periodic re-evaluation of VUS, ideally within a maximum five-year interval, until their significance in the suspected or diagnosed disease is clarified [35]. Concretely, only one study has reclassified VUS in a cohort of a SUDY population to date and identified nearly 10% of role modification five years after the first classification [27]. Nowadays, a clinically useful genetic diagnosis requires the identification of definite P/LP variants following the current American College of Medical Genetics and Genomics and the Association for Molecular Pathology (ACMG/AMP) guidelines [36], including subsequent updates. For purely diagnostic purposes, performing a new genetic test should only be performed if the previous study does not include all the known genes related to the pathology. Such genetic findings may help to establish the most plausible cause of SCA/SCD, enabling risk mitigation in genotype-positive relatives while potentially releasing genotype-negative family members from long-term surveillance [10].

## 4. Families

**Figure 2:**
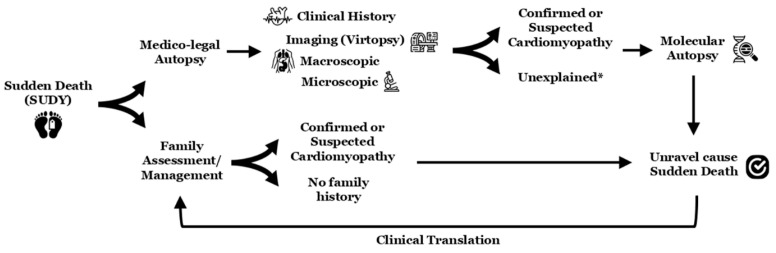
Scheme in sudden death cases (especially if young individuals). The * means potential concealed cardiomyopathy or potential cardiac channelopathy as cause of death (molecular autopsy should include all known genes associated with all inherited arrhythmogenic syndromes). SUDY, sudden unexplained death in the young.

A malignant arrhythmia leading to SCA/SCD may be the first manifestation of an IAS, including CC [1], with significant associated risk of malignant arrhythmia in first-degree relatives [37]. Unravelling the origin of SUD is crucial to providing an explanation to physicians/forensic experts as well as family members about the fatal episode. Furthermore, obtaining this information can also aid in the clinical evaluation of other family members who may be carriers of the same genetic alteration and, consequently, at risk for MA (Figure 2) [10,28,38].

Regarding genetic analysis in relatives of SCA/SCD, it is worth highlighting that familial segregation of a variant classified as P/LP according to the ACMG/AMP guidelines is widely accepted [37]. However, the management of VUS variants remains a matter of debate. One point to note is that VUS should not be clinically actionable due to their ambiguous role [33,34]; however, a VUS can be definitively reclassified as deleterious or benign, depending on new advances in this field [35]. Our experience reinforces VUS segregation, primarily helping us increase the accuracy of genetic interpretation; typically, a VUS is reclassified as benign and can therefore be eliminated as a cause of the disease [35]. This fact can alleviate anxiety in families, although clinical follow-up must be continuous, even when the precise cause of the IAS is unknown.

The current guidelines recommend a complete clinical evaluation of all surviving first-degree relatives when SCA/SCD occurs in a young individual (including asymptomatic family members). These clinical evaluations should include at least an electrocardiogram and an echocardiogram. In doubtful cases, these should be supplemented with stress tests, pharmacological tests, or high-resolution imaging tests, depending on the suspected pathology [22,23]. This clinical assessment should be complemented by genetic analysis (even if no sample is available from the deceased member) to facilitate the early identification of genetic carriers, thereby enabling the implementation of personalized preventive strategies aimed at reducing the risk of malignant arrhythmias [22,23]; despite this fact, no cause of death is identified in up to 40% of all cases due to the complexity of data interpretation [5]. The process of determining a conclusive cause of death and communicating findings to family members requires a comprehensive compilation of results, including family evaluations and follow-up at specialized centres [22,23]. Therefore, this process is multidisciplinary and involves the participation of cardiologists, pediatricians, neurologists, geneticists, genetic counsellors, and psychologists, among other professionals with experience in SCA/SCD diseases (Figure 2) [39].

Genetic counsellors play an important role not only in interpreting genetic data when translating it into clinical practice but also in explaining in detail to families what it means to be a carrier of the genetic variant, as well as the possibilities of transmitting the variant to future generations [22,37]. In this way, it is important to note that families suffering from any IAS, including CC, are characterized by variable expressivity and incomplete penetrance [14]. Therefore, an initial negative clinical assessment does not exclude the disease, and periodic follow-up is recommended, especially in genetic carriers [22,40]. It is important to emphasize that these are progressive diseases, so the appearance of clinical changes occurs over time. Thus, in families with an inconclusive diagnosis of cardiomyopathies, confirmation of the diagnosis may occur during follow-up, depending on various factors such as age, sex, the gene, and the penetrance of the genetic variant [9].

A current topic of debate concerns the frequency/duration of follow-up for family members of SUD cases [41]. A recent study with 10-year follow-up showed that most family members of SCA/SCD cases are diagnosed primarily in the first five years, suggesting the discontinuation of the follow-up of adults during this timeframe (excluding children who should receive a close follow-up). This recommendation was especially focused on those without a diagnosis, without a history of malignant cardiac events, and belonging to a family with a diagnosed IAS [42]. This represents a challenge and a high-risk practice, as the first symptom may be SCA/SCD, but we believe that it must be addressed. We recommend personalized follow-up, especially in children and young adults, with the ultimate goal of preventing future SCD events in the family [22,23].

## 5. Myocarditis

An important consideration during postmortem examination is the identification of myocardial inflammation (myocarditis), especially in the young population [43]. Myocarditis is a relatively frequent (and non-specific) feature in forensic autopsy and it may represent the cause of death; in such cases, the decease is attributed to myocarditis, most commonly of viral etiology although toxic injuries or autoimmune reactions can also cause myocardial inflammation [44,45,46,47]. Tissue inflammation superimposed on cardiomyopathy has been widely documented when macroscopic evidence of the cardiac pathology already exists (despite not always with a definite diagnosis), especially in cases of dilated cardiomyopathy and arrhythmogenic cardiomyopathy [48,49]. In SUDY cases, overlapping features of cardiomyopathy with tissue inflammation (called inflammatory cardiomyopathy) or solely myocarditis often complicates the differential diagnosis, remaining a significant challenge [46,47,50,51].

Furthermore, some studies suggest that severe myocarditis may unmask genetic predispositions to cardiomyopathy in cases with clear or suspected structural heart alterations [52,53], even with a higher mortality rate during follow-up [54]. Genetic analysis in myocarditis-related SCD was first reported in 2015. It was found that some of these patients carried P/LP variants in desmosomal genes causing cardiomyopathies in their relatives, suggesting that a genetically vulnerable myocardium may predispose to myocarditis [55]. A recent postmortem genetic study of our group identified up to a 33% positivity rate in young individuals diagnosed with myocarditis [30]. Moreover, some studies in patients suffering from complicated myocarditis (including those with severe left ventricular disfunction and ventricular arrhythmias potentially causing SCD) show a high prevalence of P/LP variants in cardiomyopathy-associated genes [56]. This also applies to family assessment, which recommends genetic testing to identify carriers of the genetic defect. These carriers of deleterious genetic variants are at risk for malignant arrhythmias, and the inflammation associated with myocarditis can trigger the fatal event. For this reason, genetic testing helps facilitate early diagnosis and the adoption of appropriate preventive therapeutic measures in each case [57].

Current evidence indicates that myocarditis represents an inflammatory stage in the phenotypic evolution of cardiomyopathies, typically arising after initial structural changes. Myocarditis in genetic cardiomyopathies contributes to earlier disease onset and is often associated with a risk of arrhythmias [58]. Therefore, in these genetically predisposed cases, myocarditis should be diagnosed and actively treated, as it is an important therapeutic target to reduce the risk of malignant arrhythmias [59]. However, SUD cases showing minimal inflammatory foci and diagnosed as myocarditis but without macroscopic abnormalities have also been reported in which the MA has identified deleterious variants associated with cardiomyopathy [60]. This suggests that the genetic alteration may create an environment conducive to the spread of an infectious agent that causes tissue inflammation leading to electrical dysfunction. Therefore, malignant arrhythmias and even SUD may be the first manifestation of CC, with inflammation being the trigger for the lethal arrhythmogenic event [55]. This tissue inflammation may even be the first evident structural alteration, especially during adolescence and young adulthood [61]. Despite these recent advances, myocarditis still has many unanswered questions, mainly in the pathophysiological mechanism involved, which makes it difficult to relate it to postmortem cases, especially if CC is suspected.

## 6. Conclusions

Nowadays, CC is an entity related to a clinically idiopathic SUD, but suspected as SCA/SCD, due to the identification by MA of a P/LP variant in one of the genes currently associated with any cardiomyopathy. CC is most often detected in young individuals, some of whom exhibit subtle, underrecognized histological alterations at autopsy, suggestive of early cardiomyopathy stages in which the malignant arrhythmia precedes structural changes. MA is strongly recommended in cases of SUD and should include a comprehensive panel of genes involved in cardiac ion channel disorders as well as cardiomyopathies. Comprehensive family assessment, including genetic segregation, is essential for an accurate interpretation of genetic data, but it also helps to identify family members who carry potentially dangerous genetic variants leading to malignant arrhythmias. Multidisciplinary teams composed of specialists are currently needed to enable a conclusive diagnosis of occult cardiomyopathy, as well as to ensure the adequate interpretation of genetic information. These teams should include forensic pathologists and cardiac histopathologists, as well as cardiologists, pediatricians, geneticists, and genetic counsellors, among others. All of these items should be implemented immediately to improve the current diagnosis yield of SUDY, identifying potential CC, and thus be able to transfer information from the forensic field to clinical practice, enabling personalized precision medicine for affected families.

## Figures and Tables

**Table 1:** Main genes associated with non-syndromic cardiomyopathies. The + sign means degree of arrhythmia or/and gross structural abnormalities. The * sign means association to cardiac channelopathies. It is important to remark that the time course toward the development of macroscopic tissue abnormalities depends on both the gene and the deleterious genetic variant. AD, Autosomic Dominant; ACM, arrhythmogenic cardiomyopathy; AR, Autosomic Recessive; DCM, dilated cardiomyopathy; HCM, hypertrophic cardiomyopathy; and RCM, restrictive cardiomyopathy.

Main Genes	Main Non-Syndromic Cardiomyopathies (Pattern of Inheritance)	Early Malignant Arrhythmias	Gross Heart Alterations
*MYBPC3*	DCM (AD)/HCM (AD)/RCM (AD)	+	++
*MYH7*	DCM (AD)/HCM (AD)/RCM (AD)	+	++
*TNNI3*	DCM (AD)/HCM (AD)/RCM (AD)	+	++
*TNNT2*	DCM (AD)/HCM (AD)/RCM (AD)	+	++
*TPM1*	DCM (AD/AR)/ HCM (AD/AR)/RCM (AD)	+	++
*ACTC1*	DCM (AD)/HCM (AD)/RCM (AD)	++	+
*MYL2*	HCM (AD/AR)	+	++
*MYL3*	HCM (AD/AR)/RCM (AD)	+	++
*ACTN2*	DCM (AD)/HCM (AD)	++	+
*PLN*	ACM (AD)/DCM (AD)/HCM (AD)	++	+
*JPH2*	DCM (AD/AR)/HCM (AD)	++	+
*FHOD3*	DCM (AD)/HCM (AD/AR)	+	++
*TNNC1*	DCM (AD)/HCM (AD)	+	++
*TTN*	ACM (AD)/DCM (AD)/RCM (AD)	+	++
*LMNA*	ACM (AD)/DCM (AD)/HCM (AD)	++	+
*RBM20*	DCM (AD/AR)	++	+
*FLNC*	ACM (AD)/DCM (AD/AR)/ HCM (AD)/RCM (AD)	++	+
*BAG3*	ACM (AD)/DCM (AD/AR)/ HCM (AD)/RCM (AD)	++	+
*DSP*	ACM (AD/AR)/DCM (AD)	+	++
*DES*	ACM (AD)/DCM (AD/AR)/ HCM (AD/AR)/RCM (AD/AR)	+	++
*TMEM43*	ACM (AD)	++	+
*SCN5A*	DCM (AD)/ *	++	+
*JUP*	ACM (AD/AR)/DCM (AD)	+	++
*VCL*	DCM (AD)/HCM (AD)	++	+
*PKP2*	ACM (AD/AR)/DCM (AD)	+	++
*DSG2*	ACM (AD/AR)/DCM (AD)	+	++
*DSC2*	ACM (AD/AR)/DCM (AD)	+	++

## Data Availability

No new data were created or analyzed in this study. Data sharing is not applicable to this article.
